# Factorial Validity and Psychometric Properties of Exercise Dependence Scale Revised Among Lebanese Exercisers

**DOI:** 10.3389/fpsyg.2022.879829

**Published:** 2022-04-14

**Authors:** Anne Deflandre, Arine Kassabian

**Affiliations:** ^1^Laboratory of Psychology, Cognition, Behavior and Communication, Rennes 2 University, Brittany, France; ^2^Department of Nutrition, Faculty of Pharmacy, Saint Joseph University of Beirut, Beirut, Lebanon

**Keywords:** measurement, validity, reliability, exercise, dependence

## Abstract

The aim of this manuscript is to discuss the reliability and the factorial, convergent and discriminant validities in measurement model of the 21-item Exercise Dependence Scale Revised (EDS-R) used for diagnosing exercise addiction. A study was conducted among 695 (364 male and 331 female) Lebanese bilingual young adults (aged 18–25) who filled the English version of this questionnaire, along with a general questionnaire. This study showed that, despite an acceptable fit index confirming the 7-factor structure and invariance across gender, exercise duration and age groups; the EDS-R lacks in reliability and convergent and discriminant validities in measurement model, due particularly to the “Reduction in other activities” subscale. The construction of the EDS-R is then to be discussed.

## Introduction

Regular exercising is often considered as a part of a healthy lifestyle (Klavestrand and Vingård, 2009; Mitchell and Barlow, 2011; Lin et al., 2015; White et al., 2017). However, in some cases, exercising may become an “unhealthy obsession” (Griffiths et al., 2005) and the main organizer of a person’s life, and even lead to exercise dependence. Exercise dependence was first conceptualized by Hausenblas and Downs as “*a craving for leisure-time physical activity, resulting in uncontrollable excessive behavior, that manifests in physiological (e.g., tolerance/withdrawal) and/or psychological (e.g., anxiety, depression) symptoms*” (Hausenblas and Downs, 2002a, p.90) and operationalized as “*multidimensional maladaptive pattern of exercise, leading to clinically impairment or distress*” (Hausenblas and Downs, 2002b, p.391).

Exercise dependence is characterized by the presence of at least three of the following criteria: tolerance, withdrawal symptoms, intention effects, lack of control, time, reduction in other activities (conflict), and continuance. These criteria were set based on the Diagnostic and Statistical Manual of Mental Disorder-IV’s (DSM-IV) criteria for substance dependence (American Psychiatric Association, 1994).

Hausenblas and Downs (2002c, p.4) defined each criterion as follows:

- Tolerance: “*either a need for increased amounts of exercise to achieve the desired effect or a diminished effect occurs with continued use of the same amount of exercise*”;
- Withdrawal: “*manifested by either the characteristic withdrawal symptoms for exercise (e.g., anxiety, fatigue) or the same (or closely related) amount of exercise is taken to relieve or avoid withdrawal symptoms*”;
- Intention Effect: “*exercise is often taken in larger amounts or over a longer period than was intended*”;
- Lack of Control: “*a persistent desire or unsuccessful effort to cut down or control exercise*”;
- Time: “*a great deal of time is spent in activities necessary to obtain exercise (e.g., physical activity vacations)*”;
- Reductions in Other Activities: “*social, occupational, or recreational activities are given up or reduced because of exercise*”;
- Continuance: *“exercise is continued despite knowledge of having a persistent or recurrent physical or psychological problem that is likely to have been caused or exacerbated by the exercise (e.g., continued running despite injury)*.”

Based on the seven aforementioned criteria, Hausenblas and Downs first proposed a 30-item “Exercise Dependence Scale” (EDS) and agreed on a 6-point Likert scale, ranging from 1 (never) to 6 (always), to evaluate each statement. They then proposed the revised and final version of the scale consisting of 21 items (7 subscales, each 3 items) rated on the same Likert scale. This version is named the “Exercise Dependance Scale—Revised” or EDS-R (Downs et al., 2004).

This frequently used scale has prompted numerous studies of structural validation using confirmatory analysis. Studies made by Downs et al. (2004), Allegre and Therme (2008, French version), Lindwall and Palmeira (2009, Swedish and Portuguese versions), Sicilia and González-Cutre (2011, Spanish version), Costa et al. (2012, Italian version), Mónok et al. (2012, Hungurian version), Parastatidou et al. (2012, Greek version), Alchieri et al. (2015, Brazilian Poruguese version), Shin and You (2015, Korean version) using confirmatory analysis with maximum likelihood estimator and by Müller et al. (2013, German version), using weighted least square, confirm the first-order seven-factor model (see Table 1). However, some items’ factor loadings are inferior to 0.70 which could be problematic according to Hair et al. (2017). The concerned items, especially those related to the “Reduction in other activities” subscale, shared more variance with its measurement error than with the construct.

**TABLE 1:** Summary of results.

Studies	Participants	Statistic procedure	Results					
			χ^2^	CFI	RMSEA	Loading	r	α
Downs et al. (2004)	140 men, 268 women 20.2 years ± 2.5	ML	–	0.96	0.06	0.56–0.96		0.67–0.93
	427 men, 428 women 21.4 years ± 2.4	ML	-	0.97	0.05	0.68–0.91	0.40–0.78	0.78–0.95
Kern (2007)	474 men, 337 women 16–60 years-old	ML^$^	748.50	0.94	0.062	0.54–0.90 0.25–0.88^£^	0.18–0.65	0.75–0.89
Allegre and Therme (2008)	402 men, 114 women 17–74 years-old	ML	470.4	0.94	0.061	–	–	0.57–0.87
Lindwall and Palmeira (2009)	95 men, 67 women 22.6 years ± 9.1^1^	ML S-B χ^2^	199.68	0.973	0.035	0.34–0.92	−0.03 to 0.83	0.55–0.89
	155 men, 114 women 26.1 years ± 8.2^2^		236.83	0.968	0.041	0.59–0.91	0.21–0.60	0.74–0.88
Sicilia and González-Cutre (2011)	271 men, 256 women 16–60 years-old	ML, boots ML, boots.^$^	489.98 615.99	0.94 0.92	0.060 0.067	0.46–0.89	0.32–0.84 0.57–0.87	0.68–0.85
Parastatidou et al. (2012)	302 men, 279 women 15–68 years-old	ML, S-B χ^2^	398.24	0.959	0.049	0.60–0.88	-	0.68–0.88
Costa et al. (2012)	262 men, 257 women 37.14 years ± 13.40	ML, S-B χ^2^	-	0.97	0.038	0.51–0.90	-	0.74–0.89
Mónok et al. (2012)	270 men, 204 women 33.2 years ± 12.1	MLR	351.9	0.938	0.049	0.45–0.88	-	0.62–0.88
Müller et al. (2013)	1611 individuals 16–60 years-old	WLS	681.80	0.99	0.040	0.87–0.96	0.79–0.94	0.83–0.93
		WLS^$^	888.37	0.99	0.050	0.89–0.96^£^		
Shin and You (2015)	247 men, 145 women 32.8 years ± 10.5	ML, S-B χ^2^	201.53	1.00	0.030	0.69–0.98	0.34–0.61	0.79–0.95
Alchieri et al. (2015)	426 men, 283 women 12–73 years-old	ML	288.21	0.95	0.04	0.35–0.94	0.08–0.70	0.66–0.84

*ML, maximum likelihood; S-B χ^2^, Satorra-Bentler scaled Chi-Square (robust to non-normality of observations); boots., bootstrapping; MLR, maximum likelihood robust (to non-normality and non-independence of observations); WLS, weighted least square; r, Inter-correlations among the seven factors; α, Cronbach’ coefficient alpha.*

*^1^Swedish sample, ^2^Portuguese sample. ^$^Seven first-order factor and one second-order factor; ^£^loadings of the first-order factors to the second order factor.*

A few studies test alternative models: Kern (2007, French version) and Müller et al. (2013) reject a one-factor model, Allegre and Therme (2008) reject a first-order six-factor model where the items of “lack of control” and “time” dimensions form a single factor and Kern (2007); Sicilia and González-Cutre (2011), and Müller et al. (2013) found a higher-order factor model (7 first-order factors and 1 second-order factor) with acceptable fit indices. Müller et al. proposed that “*the nature of this higher-order factor may reflect the unifying feature that comprehensively defines exercise dependence*” (Müller et al., 2013, p.216). Thus, the EDS-R could be considered as a one-dimensional scale.

To complete the structural analysis, Lindwall and Palmeira (2009); Sicilia and González-Cutre (2011), and Shin and You (2015) studied invariance analysis. Sicilia and González-Cutre (2011) indicated a partial invariance across age (cut-off point: 28 years-old) both for first-order seven-factor model (no significant difference between unconstrained model and model with invariant measurement weights) and higher-order factor model (no significant difference between the unconstrained model and the models with invariant measurement weights and invariant structural weights). The results of Lindwall and Palmeira (2009) suggest that the first-order seven-factor model is non-invariant across the Portuguese and Swedish samples. However, after analyzing the impact of constraints on model fit (using the Lagrange Multiplier [LM] test), the authors noted that two factor loadings were non-invariant across the samples (item 19 “I choose to exercise to get out of spending time with family/friends” and item 3 “I continually increase my exercise intensity to achieve the desired effects/benefits”). After the release of the constraints, results supported partial measurement invariance. Shin and You (2015) conclude that the first-order seven factors model is invariant across gender-groups (configural, metric, and scalar invariance). However, the aforementioned authors did not study the structural invariance across several possible subgroups (such as age, gender, or weekly exercise duration) of their studies, which would ensure that factorial structure is equivalent across the main characteristics of the studied populations.

Some studies focused on the convergent validity in measurement model and the reliability to test the homogeneity of constructs, using average variance extracted (Fornell and Larcker, 1981), construct reliability [weighted omega recommended for Bacon et al. (1995)] or also Cronbach’ coefficient alpha and on the discriminant validity in measurement model, using the examination of correlation between constructs and modification indices.

To ensure that each standardized indicator shares more variance with its latent construct than with its measurement error, Downs et al. (2004); Lindwall and Palmeira (2009), and Costa et al. (2012) calculated the average variance extracted (AVE). Lindwall and Palmeira found two subscales with an AVE inferior to 0.50 (“Lack of control”: AVE = 0.39 for the Swedish sample; “Reduction in other activities”: AVE = 0.31 for the Swedish sample; other subscales: AVE = 0.51–0.73). Costa et al. (2012) also found an AVE inferior to 0.50 for subscale “Reduction in other activities” (AVE = 0.44; other subscales: AVE = 0.53–0.73). Downs et al. (2004)^1^ reported values of AVE superior to 0.50 in both studies (0.56–0.95 in study 1 and 0.70–0.93 in study 2), however, the lowest AVE values were related to the subscale “Reduction in other activities.” Otherwise, Cronbach’s α was calculated in all the samples to study the internal consistency. Downs et al. (2004), Allegre and Therme (2008), Lindwall and Palmeira (2009, for Swedish sample), Costa et al. (2012), Mónok et al. (2012), and Parastatidou et al. (2012) reported a Cronbach’s α inferior to 0.70 [according to the recommendations of Nunnally (1978)] for the subscale “Reduction in other activities.” Other subscales presented weak internal consistency concerning “Lack of control” (Lindwall and Palmeira, 2009, for the Swedish sample; Mónok et al., 2012), “Continuance” (Mónok et al., 2012), and “Withdrawal” (Alchieri et al., 2015; see Table 1). Lindwall and Palmeira (2009, for the Swedish sample) and Costa et al. (2012) observed values of weighted Ω inferior to 0.70 for the subscale “Reduction in other activities” and Downs et al. (2004, study 1) reported a weighted Ω equal to 0.70 for the same subscale [according to the recommendations of Hair et al. (2017)].

The results of these studies highlighted problems of convergent validity and reliability in particular of the “Reduction in other activities” subscale, whatever the language version of the scale. In 2004, Downs et al. (2004, p.195) recommended future researches “*to examine the items on this subscale to determine if further item modification is necessary*.”

Concerning discriminant validity in measurement model, several studies reported high correlation (superior to 0.80) between constructs in particular those of “Reduction in other activities,” “Time,” “Lack of control,” and “Intention effects,” which could be problematic (Lindwall and Palmeira, 2009; Sicilia and González-Cutre, 2011; Müller et al., 2013).

Using modification indices, Lindwall and Palmeira (2009) reported, for the Swedish sample, a significant fit model improvement if the item 18 “I am unable to reduce how intensively I exercise” is allowed to represent the “Intentions effects” and for the Portuguese sample, if the item 12 “I think about exercise when I should be concentrating on school/work” is allowed to cross load on the “Time” and “Continuance” factors. Mónok et al. (2012) revealed that item 9 “I exercise when injured” is a complex item cross-loading on four other factors and that its elimination improves the model fit. All of the aforementioned researches revealed problems of model specification, the other researches did not undertake study of the modification indices.

Only, Downs et al. (2004) have studied, in the original version, the psychometric qualities of the EDS-R scale. The aim of the present article is to evaluate the factorial validity, reliability and the convergent and discriminant validity in measurement model of the American (English) version of 21-item EDS-R administered to non-native English-speaking students, using the Fornell and Larcker’s (1981) methodology and the recommendations of Hair et al. (2017). It was assumed, in view of the current literature, that the “Reduction in other activities” subscale would indicate, more than the six other subscales, the validity and reliability problems and would be highly correlated with one or several other subscales as “Time,” “Lack of control,” or “Intention effects.”

## Materials and Methods

### Procedures and Participants

Prior to the start of the study, the study protocol was submitted, then approved by the Ethics Committee of the University. Moreover, informed consent was obtained from each participant before inclusion in the study. The study participants were regular exercisers, recruited randomly at the university gym and the sports fields, before or after training sessions. Each Participant completed two self-administered questionnaires: a general questionnaire and the EDS-R, in the presence of a study personal, ready to clarify any misunderstood item.

A total of 695 participants were included in the study: 364 men and 331 women, aged between 18 and 25 (mean age = 21.07 years, standard deviation SD = 1.70). All participants were students at the Saint-Joseph University, Beirut (capital city of Lebanon) and regularly practiced physical exercise. Among these participants, 131 (68 men and 63 women, mean = 20.79 years, SD = 1.62) were university athletes who practiced and competed with the university team.

### Measures

#### Socio-Demographic and Weekly Exercise Duration

A self-administered general questionnaire was used to collect socio-demographic data such as gender, age, and field of studies. The questionnaire also included questions intended to estimate the weekly exercise duration of each participant. The sample reported an average of 6 h (SD = 4 h; range = 2–30 h) of physical exercising per week.

#### Exercise Dependence Scale—Revised

The English version of EDS-R (Downs et al., 2004) was administered to bilingual students who had obtained the level A (Advanced English) or B (Upper intermediate) in English proficiency tests proposed by the University. The EDS-R items, having a high level of readability and understandability, are easy to be completed by non-native speakers. Indeed, studies have shown that, in individuals with good language skills, which was the case in the present study, nativeness has no effect on the factorial structure of the measurement instrument [Young et al. (2010) for mathematics and science tests; Mehling et al. (2018) for Multidimensional Assessment of Interoceptive Awareness].

### Data Analysis

The open-access software R and package Lavaan (Rosseel, 2012) were used to perform the statistical analyses.

Preliminary analysis was conducted to check statistical assumption of normality. It revealed Skewness and Kurtosis indices ranging from −0.53 (item 3) to 1.19 (item 19) and −1.12 (item 8) to 0.51 (item 19), respectively, and an important Mardia’s coefficient of multivariate kurtosis (42.31) that allows assuming that the data come from a multivariate normal distribution.

The correlation matrix of observed variables was examined to ensure that the correlations between the same construct’s indicators are significant and superior to those between the different construct’s indicators as Fornell and Larcker (1981) recommended.

Confirmatory factor analysis (CFA) was used to test the theoretical model with correlated first-order seven-factor model (M1). The maximum likelihood method was used with Satorra-Bentler scaling correction (S-B χ^2^) to estimate the fit parameters of CFA models (Satorra and Bentler, 2001). Olsson et al. (2000) recommend the use of maximum likelihood estimator instead weighted least squares even if the data are not normally distributed and the multinormality has been controlled.

Several fit indices were analyzed: the chi square goodness-of-fit statistic (χ^2^), the root mean square error of approximation (RMSEA), the standardized root mean square residual (SRMR), the comparative fit index (CFI), and the Tucker-Lewis index (TLI). RMSEA and SRMR with respective values of 0.06 and 0.8 or less and CFI and TLI superior to 0.90 indicate a good model fit and superior to 0.95 indicate an excellent model fit (Bentler, 1990; Hu and Bentler, 1998).

Invariance analysis across gender, age (cut off at the median: 21 years old) and exercise duration (cut off at the median: 5 h) groups was used to test the equivalence of the factorial structure using three models: *model 1*: configural invariance, model with the same factorial structure on all groups; *model 2*: weak invariance or metric invariance, model with equal loadings and *model 3*: strong invariance or scalar, model with equal loadings and intercepts (Steenkamp and Baumgartner, 1998). All these parameters account for the construct validity.

To test the reliability, Cronbach’s alpha (α) and weighted omega (Ωw) were calculated. Both values superior to 0.70 indicate an acceptable validity (Nunnally, 1978; Bacon et al., 1995).

To test the convergent validity in measurement model, factor loadings of the indicators with their respective constructs were examined and AVE was calculated. Hair et al. (2017) recommended that the items’ standardized factor loadings greater than or equal to 0.70 and AVE values superior to 0.50 indicate an acceptable convergent validity (Hair et al., 2017).

To test the discriminant validity in measurement model, AVE was used in comparison to squared correlation between latent variables (Υ^2^) as well as the examination of correlation matrix between indicators (Kendall’s tau coefficient) (Fornell and Larcker, 1981; Hair et al., 2017) and the examination of LM test modification indices to find untenable equality constraints. AVE superior to Υ^2^ indicate that indicators share more variance with their construct than with another construct.

## Results

### Bivariate Analysis

Bivariate correlations analysis of same construct’s indicators revealed significant correlations (*p* < 0.001) with values ranging from 0.39 to 0.52 for the subscale “Withdrawal,” 0.33–0.44 for “Continuance,” 0.40–0.22 for “Tolerance,” 0.46–0.53 for “Lack of control,” 0.31–0.39 for “Reduction in other activities,” 0.49–0.54 for “Time,” and 0.56–0.61 for “Intention effects.” Correlation coefficients equal or higher than those mentioned were found between the indicators of different constructs, mainly between “Reduction in other activities” indicators and those of other constructs: “Time” (8 correlations ranging from 0.32 to 0.46), “Continuance” (3 correlations ranging from 0.31 to 0.32), “Lack of control” (2 correlations ranging from 0.35 to 0.36), or “Intention effects” (2 correlations ranging from 0.31 to 0.32).

### Confirmatory Factor Analysis

The confirmatory factor analysis results of correlated first-order seven-factor model (M1) indicate adequate fit indices (χ^2^ = 447.02, df = 184, *p*-value < 0.0001, CFI = 0.959, TLI = 0.939, RMSEA = 0.049, 90% CI = 0.044–0.054, SRMR = 0.035).

Seven standardized factor loadings have values inferior to 0.70 suggesting that “Withdrawal,” “Continuance,” “Lack of control,” and “Reduction in other activities” are not well-determined by some of their indicators (Table 2).

**TABLE 2:** Standardized factor loadings and internal consistency reliabilities.

Items	Factor loading (M1)
*Withdrawal*	**AVE** = 0.538; **α** = 0.771; **Ω_*w*_** = 0.793
Item 1	0.616
Item 8	0.777
Item 15	0.793
*Continuance*	**AVE** = 0.505; **α** = 0.740; **Ω_*w*_** = 0.795
Item 2	0.650
Item 9	0.610
Item 16	0.848
*Tolerance*	**AVE** = 0.569; **α** = 0.792; **Ω_*w*_** = 0.811
Item 3	0.697
Item 10	0.831
Item 17	0.727
*Lack of control*	**AVE** = 0.577; **α** = 0.804; **Ω_*w*_** = 0.812
Item 4	0.681
Item 11	0.781
Item 18	0.809
*Reduction*	**AVE** = 0.411; **α** = 0.675; **Ω_*w*_** = 0.682
Item 5	0.690
Item 12	0.655
Item 19	0.571
*Time*	**AVE** = 0.624; **α** = 0.833; **Ω_*w*_** = 0.832
Item 6	0.786
Item 13	0.792
Item 20	0.790
*Intention effects*	**AVE** = 0.672; **α** = 0.860; **Ω_*w*_** = 0.861
Item 7	0.820
Item 14	0.837
Item 21	0.800

*AVE, Average Variance Extracted; α, Cronbach’s Alpha; Ω_w_, Weighted Omega.*

Table 3 shows the correlations among the seven factors. A very high correlation is observed between “Reduction in other activities” and “Time” (0.907).

**TABLE 3:** Inter-correlations between the seven latent variables.

Factors	1	2	3	4	5	6	7
1. Withdrawal	–						
2. Continuance	0.430	–					
3. Tolerance	0.405	0.416	–				
4. Lack of control	0.440	0.524	0.599	–			
5. Reduction	0.478	0.645	0.496	0.702	–		
6. Times	0.508	0.530	0.559	0.749	0.907	–	
7. Intention	0.494	0.480	0.533	0.666	0.635	0.732	–

### Multigroup Invariance Testing

Table 4 summarizes the results of the full invariance analysis across gender, age, and duration groups. Configural model shows an adequate fit about gender-groups (χ^2^ = 658.77, df = 336, *p*-value < 0.0001, CFI = 0.945, TLI = 0.931, RMSEA = 0.053, 90% CI = 0.047–0.058, SRMR = 0.042), age-groups (χ^2^ = 619.30, df = 336, *p*-value < 0.0001, CFI = 0.951, TLI = 0.939, RMSEA = 0.049, 90% CI = 0.044–0.055, SRMR = 0.042), and duration-groups (χ^2^ = 634.04, df = 336, *p*-value < 0.0001, CFI = 0.939, TLI = 0.924, RMSEA = 0.051, 90% CI = 0.045–0.056, SRMR = 0.044). Configural invariant is supported across groups.

**TABLE 4:** Goodness-of-fit indexes for models multi-sample CFA models.

	*df*	χ*^2^*	Δχ*^2^*	Δ*CFI*	Δ*RMSEA*	*p*
**Gender-groups**						
Model 1	336	832.17				
Model 2	350	859.22	27.051	0.002	0.000	0.019
Model 3	364	897.30	38.081	0.004	0.000	***
**Age-groups**						
Model 1	336	781.61				
Model 2	350	798.58	16.964	0.000	0.001	0.253
Model 3	364	814.20	15.620	0.000	0.001	0.337
**Duration-groups**						
Model 1	336	798.78				
Model 2	350	811.81	13.024	0.001	0.001	0.524
Model 3	364	856.00	44.188	0.005	0.001	***

df, degrees of freedom; χ^2^, chi-square; Δχ^2^, difference of chi-square; ΔCFI, difference of robust comparative fit index; ΔRMSEA, difference of robust root-mean square error of approximation; ***p < 0.00001.

No significant differences were found between the *model 1* (configural invariance) and the *model 2* (metric invariance) across age and duration groups and the *model 2* and the *model 3* (scalar invariance) across age-groups. The lack of significant difference between the two models is the minimal criterion for accepting the construct-level metric invariance (Byrne et al., 1989). The metric invariance is established across duration-groups and the scalar invariance is established across age-groups. Based on the acceptable ΔCFI, multigroup invariance is supported (Cheung and Rensvold, 2002).

### Reliability and Convergent Validity in Measurement Model

Cronbach’s α, Ω_*w*_, and AVE are reported in Table 2. The subscale “Reduction in other activities” has an AVE, α, and Ω_*w*_ values inferior to the recommendations.

### Discriminant Validity in Measurement Model

The examination of the correlation matrix between indicators (see Section “Bivariate Analysis”) shows higher correlations between the “Reduction in other activities” indicators and several indicators of other constructs than between the indicators of this subscale, which is considered as a problem. Fornell and Larcker (1981) indicated that “*discriminant validity is exhibited only if all the correlations in R_*xx*_ and R_*yy*_ (measurement) are statistically significant and each of these correlations is larger than all correlations in R_*xy*_*,” R_*xx*_ and R_*yy*_ referring to the correlation matrix between indicators of same construct and R_*xy*_ referring to the correlation matrix between indicators of different construct. Moreover “Reduction in other activities” and “Time” constructs are correlated strongly (Table 3).

Fornell and Larcker (1981) proposed to pay a particular attention to the squared gamma measuring the variance shared by two constructs. The requirements for discriminant validity are fully satisfied if squared gamma is inferior to the average variance extracted from each construct. In the case of “Reduction in activities” and “Time,” squared gamma was superior to the respective AVE. These results show that a fairly strong overall relationship exists between these two constructs.

The analysis of modification indices did not reveal the presence of complex items with salient factor loadings with other constructs.

## Discussion

The aim of this study was to examine the factorial validity, reliability and the convergent and discriminant validity in measurement model of the American (English) version of 21-item EDS-R administered to non-native English-speaking students, and to test the hypothesis concerning the problematic nature of the “Reduction in other activities” subscale. The results indicate that, despite acceptable fit indexes confirming the 7-factor structure and multigroup invariance of the 21-item EDS-R, the reliability, convergent, and discriminant validity of this tool are problematic, particularly due to the subscale “Reduction in other activities.”

Previous studies have also identified concerns related to the convergent validity and reliability of the subscale “reduction in other activities.” A factor loading inferior to 0.70 (Downs et al., 2004; Kern, 2007; Lindwall and Palmeira, 2009; Sicilia and González-Cutre, 2011; Costa et al., 2012; Mónok et al., 2012; Parastatidou et al., 2012; Alchieri et al., 2015) and an average variance extracted inferior to 0.50 and Cronbach’s alpha inferior to 0.70 (Downs et al., 2004; Allegre and Therme, 2008; Lindwall and Palmeira, 2009; Sicilia and González-Cutre, 2011; Mónok et al., 2012; Parastatidou et al., 2012) have been established for this subscale.

A few studies have reported a discriminant validity in measurement, and demonstrated that the latent variable measure is very specific. The results of this study show higher bivariate correlations between items of different subscales (“Time” × “Reduction in other activities”) than between items within the subscale of “Reduction in other activities.” The observed correlation matrix between items has never been reported in the examined studies, which does not allow us to detect similar results. Moreover, the variance shared by these two constructs is superior to the average variance extracted from each construct, and the very high observed correlation between these factors (0.907) (Hinkle et al., 2003) shows that the “Reduction in other activities” items report information already contained in “Time” items, or inversely. In this study, the discriminant validity is problematic; each item does not operate as an indicator of a distinct construct. Lindwall and Palmeira (2009) and Müller et al. (2013) have also reported high correlations between the factors “Time” and “Reduction in other activities” (superior to 0.80). Sicilia and González-Cutre (2011) and Müller et al. (2013) have mentioned other high correlations between the factors “Time” and “Lack of control” or “Intention effects” and “Time” as is the case of the results of this study, where there is also a high correlation between the factors “Reduction in other activities” and “Lack of control.” These authors have also reported a high correlation between the factors “Reduction in other activities” and “Intention effects.” Based on Anderson and Gerbing’s (1988) additional recommendations, Parastatidou et al. (2012) found acceptable discriminant validity values. Similarly, Sicilia and González-Cutre (2011), not observing “*very high correlation*” among the subscales, ended up with good discriminant validity. However, by examining the results of these authors and in view of Fornell and Larcker’s (1981) recommendations, it appears that the information contained in the “Reduction in other activities” subscale is redundant with those of the “Intentions effects” subscale (Υ^2^ > AVE for each subscale). The other studies did not mention results on discriminant validity. Using a criterion Υ^2^ > AVE (for each subscale) that can be calculated from the available information, the results of Downs et al. (2004); Lindwall and Palmeira (2009), Alchieri et al. (2015), and Shin and You (2015) did not allow the detection of flagrant discriminant validity problems. However, Fornell and Larcker (1981) pointed out that this criterion should not be the only one to conclude on this validity. The results of this study did not reveal the presence of complex items as Lindwall and Palmeira (2009) and Mónok et al. (2012). In this direction, it seems interesting to use several criteria to detect possible problems of discriminant validity.

Problems of convergent and discriminant validity and/or reliability have been reported by different studies involving, in particular, the subscale “Reduction in other activities” as demonstrated in the present study as well. Downs et al. (2004) have already pointed out the weakness of these items and proposed to reformulate them. Nonetheless, would not it be better to delete them? Indeed, “Reduction in other activities” is undoubtedly the consequence of increased time investment in exercise and should not be considered as a separate symptom. The criterion “Reduction in other activities” (DSM-IV, 1994) refers to a time-sharing conflict between the object of addiction (referring to substance use in the 1994 version of the DSM; and to exercise practice in the present study) and other activities: “virtually all of the person’s daily activities revolve around the substance” (DSM-IV, 1994).

The wording of the “Reduction in other activities” items must also be questioned. Indeed, is exercise dependence a matter of preference or choice, as suggested in item 5 “I would rather exercise than spend time with family/friends” and item 19 “I choose to exercise so that I can get out of spending time with family/friends “? These ideas of choice or preference contradict to that of “*uncontrollable excessive behavior*” (Hausenblas and Downs, 2002a). To measure the conflict between activities, the items could be formulated, as: I exercise so much that I cannot spend time on family activities/friendly activities/occupational activities or studies. An indirect wording could also be used, such as “my exercise practice is in conflict with my family activities,” to remove the idea of time and perhaps weaken the problematic correlation between this scale and that of “Time.”

In the same way, the substance dependence criteria of “lack of control” or “intention effects” do not refer to the notion of time. On the contrary, in the case of exercise dependence, desired effects are proportional to the time spent exercising and lack of control and intention effects are related to the notion of time (e.g., “I exercise longer than,” “unable to reduce how long”). Moreover these two constructs are very close. As such, is not “to perform an exercise longer than expected” the expression of lack of control?

In the Hausenblas and Downs (2002a) definition, one central parameter was retained: uncontrollability of the exercise. Uncontrollability could cover time investment to exercise, reduction in other activities, intention effects and lack of control, factors that are often highly correlated. As a result, clinical use of the EDS-R could lead to over-detection of individuals at-risk or non-dependent-symptomatic. However, it is important to emphasize that it is not the time spent exercising that is symptomatic of the dependence, but it’s the lack/loss of control of this time, at the risk to over-detect of dependent individuals (Freimuth et al., 2011; Egorov and Szabo, 2013; Landolfi, 2013).

The DSM-V (American Psychiatric Association, 2013) combines time, intention effects and lack of control within an overall grouping: impaired control and reduction in other activities within social impairment grouping that include the negative social consequences of substance use that do not appear in the DSM IV criteria of substance dependence. Otherwise, the transposition of substance dependence criteria to those of the exercise dependence is problematic. Indeed, the amount of substance refers, in the case of exercise, to the duration (time), frequency and intensity of the exercise practice. The same term is thus used to define and measure an amount and a criterion.

This transposition is not so obvious as suggested by the lack of consensus on the criteria of exercise dependence highlighted in the DSM–V (2013) and its non-inclusion. This manual has introduced non-substance-related disorders (gambling disorder, formerly pathological gambling, and internet gaming disorder), has removed the distinction between dependence and abuse (in favor of a range of severity based on the number of symptom-criteria endorsed) and has integrated negative social consequences (formerly, substance abuse criterion) and proposed the overall groupings of criteria (impaired control, social impairment, risky use, and pharmacological criteria). These advances should probably permit to clarify the conceptualization of exercise dependence by associating behavioral components with the criteria of substance dependence as suggested by Allegre et al. (2006) and to review the items wording containing the idea of time.

## Conclusion

This study intended to check the construct, the discriminant and convergent validities in measurement model, and the reliability of the 21-item EDS-R, in the most complete way possible, using the specific recommendations of Fornell and Larcker (1981) and Hair et al. (2017). The objective of this study was attained, and the results showed that the “Reduction of other activities” subscale is particularly flawed and requires a thorough revision. It should be noted that other measurements of validity and reliability could have been realized, other recommendations could have been used, and other statistical analyses could have been carried out. However, considering warnings from literature sources, it seemed relevant to study these criteria before undertaking further analyses, revisions or translations of the scale, in order to ensure that the EDS-R measures more the constructs it is supposed to measure.

## Data Availability Statement

The raw data supporting the conclusions of this article will be made available by the authors, without undue reservation.

## Ethics Statement

The studies involving human participants were reviewed and approved by the Ethics Committee of the Saint Joseph University. The patients/participants provided their written informed consent to participate in this study.

## Author Contributions

AD: manuscript writing. AK: proofreading and data collection. Both authors contributed to the article and approved the submitted version.

## Conflict of Interest

The authors declare that the research was conducted in the absence of any commercial or financial relationships that could be construed as a potential conflict of interest.

## Publisher’s Note

All claims expressed in this article are solely those of the authors and do not necessarily represent those of their affiliated organizations, or those of the publisher, the editors and the reviewers. Any product that may be evaluated in this article, or claim that may be made by its manufacturer, is not guaranteed or endorsed by the publisher.

## Footnotes

1 However, in this study, the reported AVE values may not be correct. AVE’ calculation from factor loadings show much lower values.
